# Clinical advances and challenges in dose constraints for organs at risk in proton therapy: A systematic review of the last decade's literature

**DOI:** 10.1002/pro6.70020

**Published:** 2025-07-21

**Authors:** Jinghao Duan, Cheng Tao, Shizhang Wu, Chengqiang Li, Jinhu Chen, Tianyuan Dai, Yunyi Fan, Tong Bai, Xiangjuan Meng, Tian Kong, Jian Zhu

**Affiliations:** ^1^ Department of Radiation Physics and Technology Shandong Cancer Hospital and Institute, Shandong First Medical University and Shandong Academy of Medical Sciences Jinan China; ^2^ Shandong Provincial Key Medical and Health Laboratory of Pediatric Cancer Precision Radiotherapy (Shandong Cancer Hospital) Jinan China; ^3^ Department of Clinical Lab Shandong Cancer Hospital and Institute Shandong First Medical University and Shandong Academy of Medical Sciences Jinan China; ^4^ Disease Control and Prevention Institute of China Railway Jinan Bureau Group Co., Ltd Jinan China

**Keywords:** dose constraints, organs at risk, proton radiation therapy, radiotherapy

## Abstract

Proton beam therapy has demonstrated significant clinical efficacy across multiple malignancies, primarily attributed to its distinct physical dose deposition characteristics. However, clinical implementation remains constrained by resource limitations and accumulated experience, particularly regarding radiation tolerance thresholds for organs at risk (OARs). Unlike photon‐based radiotherapy where consensus guidelines like QUANTEC have been established, standardized dose constraints for proton therapy require further validation. This systematic review synthesizes decade‐long evidence from peer‐reviewed literature and clinical guidelines, critically evaluating current understanding of OARs tolerance in proton therapy. The comprehensive analysis aims to inform clinical decision‐making and protocol development for emerging proton therapy.

## Graphical abstract

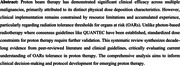

## 1 INTRODUCTION

Proton therapy employs particle accelerators to propel protons into high‐energy states, allowing precise three‐dimensional control of beam energy and directional modulation. This capability facilitates the characteristic Bragg peak phenomenon, where maximum energy deposition occurs at a predetermined tissue depth. The sharp distal dose gradient inherent to proton beams enables concentrated tumor irradiation while substantially sparing distal healthy tissues from radiation exposure. Compared to X‐rays, protons exhibit a relative biological effectiveness (RBE) of approximately 1.1, indicating a 10% increase in biological efficacy. This value, widely adopted in standard proton treatment planning, represents a simplified assumption derived from aggregated experimental data.^1^, ^2^ However, proton RBE is not constant and varies depending on several factors, including energy and depth dependence, tissue‐type specificity, and dose fractionation regimens. Notably, at the distal edge of the Bragg peak, where ionization density increases significantly, localized RBE values may rise to 1.5–1.7 due to the induction of more complex DNA damage patterns. Additionally, differential radiation sensitivity across tissues (e.g., tumors versus normal tissues) further modulates RBE outcomes. Intriguingly, single high‐dose fractions may demonstrate lower RBE values compared to fractionated irradiation schemes, highlighting the importance of dose delivery parameters in biological effectiveness assessments.^3^, ^4^ Therefore, proton therapy exhibits distinct dose constraints compared to conventional photon therapy due to fundamental differences in their physical dose deposition patterns and biological effectiveness.

According to the Particle Therapy Co‐Operative Group (PTCOG) 2023 global census, over 410,000 patients had received particle therapy worldwide, with proton modalities constituting approximately 85% (n≈350,000) of total treatments. Despite this progressive clinical adoption, proton therapy utilization remains orders of magnitude below conventional photon‐based radiotherapy. This disparity has resulted in concentrated expertise within specialized proton therapy centers and a paucity of multi‐institutional clinical data. Crucially, proton radiotherapy currently lacks standardized organs‐at‐risk (OARs) dose constraints comparable to photon‐specific QUANTEC guidelines, with existing protocols predominantly relying on single‐institution experiences rather than evidence‐based consensus parameters for normal tissue complication probability modeling.

## 2 METHODOLOGY

This review synthesizes advancements in proton radiotherapy dosimetry from January 2014 to Februay 2024, focusing on radiation‐induced toxicities and OARs constraints across five clinical domains: adult central nervous system tumors, head and neck carcinomas, thoracic malignancies, abdominopelvic neoplasms, and pediatric cancers. A systematic literature search was conducted using Web of Science, PubMed, and the Wanfang Database with the following combined English/Chinese keywords: “Proton therapy,” “Proton radiation oncology,” “Proton radiotherapy”, “Complication”, “Toxicity”, “Tolerance”, “Organs at risk” and “Normal tissue”.

For the literatures, studies were selected based on the PICOS framework. **Exclusion criteria comprised case reports, non‐peer‐reviewed publications, duplicate datasets or overlapping cohorts, and studies with insufficient data**. Initial screening ** yielded** 263 relevant articles (227 English, 36 Chinese). **Following** critical appraisal, 32 articles (30 English, 2 Chinese) were **retained**. Supplementary references were extracted from the Springer‐published monograph *Target Volume Delineation and Treatment Planning for Particle Therapy* and educational section from recent PTCOG conferences. This review aims to assist clinicians, medical physicists, and researchers in optimizing proton therapy planning by balancing tumor control and potential toxicities. Unless explicitly stated, all dose‐volume constraints pertain to adult populations in this paper. Secondly, proton doses are expressed in Gy (RBE) to standardize biological effectiveness.^5^

## 3 OARS DOSE CONSTRAINTS

### 3.1 Central Nervous System (CNS) tumor

Common toxicities associated with proton therapy for CNS malignancies include impairment of visual or auditory function, endocrine disorders secondary to pituitary dysfunction, cerebral necrosis, epilepsy induced by temporal lobe necrosis, secondary malignant neoplasms, cutaneous erythema/alopecia, and persistent fatigue. The European Particle Therapy Network (EPTN) convened a panel of 20 radiation oncology experts to systematically review existing literature on proton therapy for neurological tumors, culminating in consensus recommendations for dose constraints to OARs as summarized in Table 1.^6^ These consensus guidelines provide valuable reference points for clinicians engaged in proton radiotherapy for CNS malignancies. However, the current recommendations exhibit limitations in precise dose‐toxicity correlations due to restricted data availability, the paucity of large‐scale clinical trials, and predominant reliance on single‐institutional studies.

**TABLE 1:** Recommended dose constraints for organs at risk in EPTN

OARs	Dose constraint EQD2	Toxicity
Brain	V _60 Gy_ ≤ 3 cc	Symptomatic brain necrosis
Brainstem	Surface D _0.03 cc_ ≤ 60 Gy Interior D _0.03 cc_ ≤ 54 Gy	Permanent cranial neuropathy or necrosis
Chiasm & Optic nerve	D _0.03 cc_ ≤ 55 Gy	Optic neuropathy
Cochlea	D _mean_ ≤ 45 Gy D _mean_ ≤ 32 Gy	Hearing loss Tinnitus
Cornea	D _0.03 cc_ ≤ 50 Gy	Erosion/ulceration
Hippocampus	D _40%_ ≤ 7.3 Gy	Memory loss
Lacrimal gland	D _mean_ ≤ 25 Gy	Keratoconjunctivitis sicca
Lens	D _0.03 cc_ ≤ 10 Gy	Cataract
Pituitary	D _mean_ ≤ 45 Gy D _mean_ ≤ 20 Gy	Panhypopituitarism Growth hormone deficiency
Retina	D _0.03 cc_ ≤ 45 Gy	Loss of vision
Skin	D _0.03 cc_ ≤ 25 Gy	Permanent alopecia

*Note*: EQD2 = equivalent dose in 2 Gy per fraction; D _3 cc_ = dose to 3 cc of structure/organ; D _0.03 cc_ = near maximum dose to 0.3 cc of structure/organ; D _mean_ = mean dose; D _40%_ = mean dose to 40% of the volume of both hippocampi.

A phase II clinical trial investigating craniospinal irradiation for metastatic disease reported detailed normal tissue dose constraints.^7^ The results demonstrated comparable rates of grade 3 or higher toxicities between proton therapy and photon‐based radiotherapy, with no significant difference observed between the two modalities. Palma et al.^8^ identified scalp doses of 21 Gy (RBE) and 25 Gy (RBE) as optimal predictors for acute and late‐onset alopecia, respectively, while establishing D2% with a threshold of 48 Gy (RBE) as the sole dosimetric variable predictive of permanent alopecia. In a separate analysis, Dutz et al.^9^ determined three key dosimetric parameters as prognostic indicators for acute cutaneous toxicities: V35 Gy (RBE) for erythema (grade ≥1), D2% for alopecia (grade ≥1), and D5% for clinically significant alopecia (grade ≥2).

It is noteworthy that while hippocampal sparing has been recognized as crucial for neurocognitive preservation and dose constraints have been established through EPTN consensus, critics argue that the clinical significance of hippocampal dose limitation remains incompletely quantified. Specifically, current dose‐volume‐response relationships lack sufficient precision to formulate definitive clinical guidelines.^10^ Furthermore, although cranial radiotherapy may increase the risk of long‐term neurocognitive impairment, the magnitude of this risk remains uncertain.^10^

### 3.2 Head and neck tumor

Common toxicities associated with proton therapy for head and neck malignancies encompass dysphagia, mucositis, xerostomia, alterations in taste perception and appetite, nausea/vomiting, cutaneous inflammation with ulceration, dehydration, weight loss, fatigue, carotid artery blowout syndrome (meriting particular attention in re‐irradiation cases), hoarseness, otitis media, dental caries, esophageal stricture, osteoradionecrosis, unilateral/bilateral visual or auditory impairment, and secondary malignant neoplasms. The Memorial Sloan Kettering Cancer Center has established OARs dose constraints for proton radiotherapy in oropharyngeal carcinoma, as detailed in Table 2.^11^

**TABLE 2:** Oropharyngeal cancer dose constraints guidelines at Memorial Sloan Kettering Cancer Center

OARs	Dose Constraint
Oral cavity	Mean dose <35–40GyE
Spinal cord	Dose to 0.1 cc < 50 Gy RBE^a^
	Surface max < 64 Gy RBE^b^
Brainstem	Dose to 0.05 cc < 60 Gy RBE^a^
	Core max < 53 Gy RBE
	Surface max < 64 Gy RBE^b^
Cochlea^c^	Max dose < <50 Gy RBE
Parotid	Mean dose < 25 Gy RBE ALARA
Larynx	Mean dose < <35 Gy RBE

^a^ For plans with prescription dose ≤60 Gy RBE;

^b^ Isodose line may touch structure surface;

^c^ If ipsilateral hearing is absent, contralateral cochlea constraint is <35 Gy RBE

The OARs dose constraints for proton radiotherapy in paranasal sinus tumors, as recommended in the monograph *Target Volume Delineation and Treatment Planning for Particle Therapy* co‐authored by Nancy Y. Lee et al.,^11^ are presented in Table 3.

**TABLE 3:** Dose‐volume histogram (DVH) planning objectives for sinonasal proton therapy plans

OARs	DVH point	Limit	Minor deviation	Major deviation
Brain stem	Absolute dose at 0.1 cc	<55GyE	55≤D_0.1cc_<64GyE	D_0.1cc_≥64GyE
	Maximum absolute dose	<60GyE	60≤D_max_<67GyE	D_max_≥67GyE
Brain stem surface	Absolute dose at 0.1 cc	<55GyE	55≤D_0.1cc_<64GyE	D_0.1cc_≥64GyE
Brain stem core	Absolute dose at 0.1 cc	<50GyE	50≤D_0.1cc_<60GyE	D_0.1cc_≥60GyE
Spinal cord	0 Absolute dose at 0.1 cc	<50GyE	0≤D_0.1cc_<55GyE	D_0.1cc_≥55GyE
Optic chiasm	Absolute dose at 0.1 cc	<55GyE	55≤D_0.1cc_<60GyE	D_0.1cc_≥60GyE
	Maximum absolute dose	<57GyE	57≤D_max_<62GyE	D_max_≥62GyE
Optic nerve	Absolute dose at 0.1 cc	<55GyE	55≤D_0.1cc_<60GyE	D_0.1cc_≥60GyE
Retina	Absolute dose at 0.1 cc	<50GyE	50≤D_0.1cc_<60GyE	D_0.1cc_≥60GyE
Larynx	Mean absolute dose	<36GyE	‐	D_mean_≥36GyE
Cochlea	Mean absolute dose	<36GyE	36≤D_mean_<45GyE	D_mean_≥45GyE
Parotid	Mean absolute dose	<26GyE	D_mean_≥26GyE	‐
Submandibular gland	Mean absolute dose	<40GyE	D_mean_≥40GyE	‐
Cervical esophagus	Mean absolute dose	<50GyE	D_mean_≥50GyE	‐
Oral cavity	Mean absolute dose	<36GyE	D_mean_≥36GyE	‐
	Relative volume at 20Gy	<10%	V20≥10%	‐
Temporal lobe	Absolute volume at 74Gy	<2cc	V74≥2cc	‐
Hippocampus tail	Mean absolute dose	<20GyE	D_mean_≥20GyE	‐
Hippocampus head	Mean absolute dose	<5GyE	D_mean_≥5GyE	‐
Pharyngeal constrictors	Mean absolute dose	<50GyE	50≤D_mean_<60GyE	D_mean_≥60GyE
Lacrimal gland	Mean absolute dose	<34GyE	34≤D_mean_<41GyE	D_mean_≥41GyE
Hypothalamus	Mean absolute dose	<5GyE	D_mean_≥5GyE	‐
Pituitary	Mean absolute dose	<30GyE	D_mean_≥30GyE	‐
Mandible	Mean absolute dose	<40GyE	‐	D_mean_≥40GyE
	Relative volume at 70Gy	<10%	‐	V70≥10%
Brain	Absolute volume at 74Gy	<2cc	V74≥2cc	‐
Lens	Maximum absolute dose	<15GyE	D_max_≥51GyE	‐

Beddok et al.^12^ conducted a retrospective analysis of toxicity response in 55 head and neck cancer patients who underwent re‐irradiation with intensity‐modulated radiation therapy (IMRT) or proton therapy, from which they proposed dose constraints of 36.7 Gy (RBE) for pharyngeal constrictors and 20.5 Gy (RBE) for oral cavity structures. For re‐irradiation cases in head and neck malignancies, the monograph *Target Volume Delineation and Treatment Planning for Particle Therapy* ^11^ provides comprehensive documentation of radiation‐induced complications along with their corresponding dose references, as outlined in Table 4.

**TABLE 4:** Dose volume constraints for normal critical structures in head and neck reirradiation

OARs	To	Total dose	Comments
Cord	Core max	53GyE	Max from current treatment
	Surface max	64GyE	
	Dose to 0.1cc	70GyE	Max from all treatments (past and present)
Optic Chiasm	Dose to 0.05cc	60GyE	Max from current treatment
	Mean dose	58GyE	
	Dose to 0.05cc	70GyE	Max from all treatments (past and present)
Optic Nerve	Dose to 0.05cc	60GyE	Max from current treatment^a^
	Dose to 0.05cc	70GyE	Max from all treatments (past and present)^a^
Brainstem	Surface max dose	64GyE	Max from current treatment; Core defined as 3mm diameter central structure within
	Core max dose	53GyE	
	Dose to 0.05cc	70GyE	Max from all treatments (past and present)
Cochlea	Max dose	55GyE	ALARA; aim for this max from all treatments (past and present)^a^
Retina	Dose to 0.05cc	70GyE	
Lacrimal Gland	Mean dose	50GyE	
Lens	Max dose	25GyE	
Parotid	Mean dose	26GyE	
Oral Cavity	Mean dose	40GyE	
Mandible		No hot spots	ALARA; aim for this max from all treatments (past and present)
Mandible not in PTV	0.05cc	70GyE	
Branchial Plexus	D95	65GyE	
	Max dose	70GyE	
Esophagus	75Gy<1.5cc of partial circumference		
Submandibular Gland		39GyE	
Larynx	70GyE	0.05 cc	

^a^ one side may be exceeded if the contralateral side is functional; ALARA—As low as reasonably achievable.

### 3.3 Thorax tumor

Common complications following proton therapy for lung cancer encompass radiation pneumonitis, radiation esophagitis, cardiac arrhythmias, ischemic heart disease, acute/chronic heart failure, and valvular injury.^13^ Patients undergoing thoracic re‐irradiation with protons warrant heightened vigilance for complications including pneumothorax, radiation pneumonitis, bronchial obstruction, tracheoesophageal fistula, and tracheoesophageal stenosis.^14^ The University of Texas MD Anderson Cancer Center's recommended OARs dose constraints for conventionally fractionated proton radiotherapy in lung cancer are summarized in Table 5. ^11^ For twice‐daily fractionation regimens, this institution specifies a maximum spinal cord dose limit of 40 Gy (RBE) while maintaining other constraints equivalent to conventional fractionation.^11^ Furthermore, in proton therapy for locally advanced non‐small cell lung cancer, Harris et al.^15^ demonstrated significant correlations between radiation pneumonitis and dosimetric parameters spanning V35‐V50, beyond the conventional V20 and mean lung dose (Dmean). Notably, V40 ≤23% emerged as a distinct predictive parameter, contrasting with photon‐based radiotherapy paradigms. Their proposed constraints include V20 ≤35%, V35 ≤25%, V40 ≤23%, V50 ≤19%, and Dmean ≤20 Gy (RBE).

**TABLE 5:** Dosimetric constraints for proton therapy in standard fractionated radiation delivered once daily

OARs	Dose constraint
Spinal cord	Maximum dose ≤45 Gy (RBE)
Heart	V30 ≤ 45 Gy (RBE), mean dose <26 Gy (RBE)
Esophagus	Mean dose < 34 Gy (RBE), V50 < 50%
Total lung	Mean dose < 20 Gy (RBE), V20 < 35%
Kidney	20 Gy (RBE) < 33% of bilateral kidney
Liver	V30 ≤ 40%

Lymphoma patients who achieve long‐term survival following proton radiotherapy face substantial risks of developing complications including cardiovascular diseases, hypothyroidism, pulmonary fibrosis, emphysema, chronic pneumonia, esophagitis, and secondary malignancies.^16^ The International Lymphoma Radiation Oncology Society (ILROG) has established comprehensive dose constraints for OARs in proton radiotherapy for adult lymphoma patients (Table 6),^17^ with particular emphasis on cardiac substructures, breast tissue, and age‐specific dose limitations.

**TABLE 6:** Acceptable dose, volume, and field considerations in proton therapy recommended by International Lymphoma Radiation Oncology

OARs	Ideal	Optimize technique	Optimize field (consider field reduction)	Unacceptable	Avoid maximum dose landing in
Heart: left ventricle, coronary arteries, valves	Mean<5 GyE	Mean<5‐15GyE	Mean>15 GyE	Mean>30 GyE	Coronary vessels
Breast (age dependent)^a^	Mean<4 GyE	Mean<4‐15GyE	Mean>15 GyE	Mean>30 GyE	Glandular tissue
Lung	V_5_<55%, V_20_<30% Mean<10 GyE	V_5_<55‐60% Mean<10‐13.5GyE	‐	V_5_>60%, Mean>13.5 GyE	‐
Thyroid	V_25_<62.5%	V_25_<62.5%	‐	‐	Whole thyroid

^a^ The importance of adhering to breast‐dose restrictions is inversely related to patient age.

Emerging evidence in proton radiotherapy for breast cancer highlights the necessity of monitoring rib fracture risk alongside established complications including radiation pneumonitis, cutaneous inflammation, cardiotoxicity, pain, and fatigue.^18^, ^19^ Comparative analyses reveal that proton therapy utilizing 1–2 beam portals may induce more severe dermatologic toxicities than photon‐based radiotherapy, though toxicity profiles substantially improve with 2–4 beam configurations.^20^ The Breast Cancer Subcommittee of the PTCOG consensus recommends constraining the mean dose to contralateral breast to 1 Gy (RBE), a parameter particularly crucial for younger women below 40 years of age.^21^ During the PTCOG 2018 Educational Session, Professor Shannon from Massachusetts General Hospital presented institution‐specific dose constraints for breast proton therapy: esophageal maximum dose ≤40 Gy (RBE); thyroid doses maintained as low as reasonably achievable (ALARA) without absolute constraints; ipsilateral lung V20 <15‐18 Gy (RBE); cardiac parameters including maximum dose ≤3 Gy (RBE) to left anterior descending coronary artery, ≤5 Gy (RBE) to left ventricle, and mean heart dose <0.5‐1 Gy (RBE).^22^

Proton therapy for esophageal carcinoma is associated with acute toxicities including esophagitis/ulceration, anastomotic leakage, dermatitis, nausea, vomiting, fatigue, and leukopenia, while late complications may manifest months to years post‐treatment, encompassing severe cardiac, pulmonary, renal, and gastrointestinal toxicities.^23^ A pooled analysis of proton therapy for locally advanced esophageal squamous cell carcinoma established the following organ‐at‐risk dose constraints: normal lung V20 Gy (RBE) <20%, cardiac V30 Gy (RBE) <46% with a mean dose <26 Gy (RBE), and spinal cord maximum point dose <48 Gy (RBE).^24^

Patients undergoing proton therapy for mesothelioma face risks of complications commonly associated with proton therapy for other thoracic malignancies. The PTCOG has established consensus guidelines for OARs dose constraints in mesothelioma proton radiotherapy, as delineated in Table 7.^25^

**TABLE 7:** Dosimetric constraints recommendations for Mesothelioma proton plan with conventional fractionation

OARs	Dosimetric constraints
Contralateral lung	Mean dose < 1.5 Gy; V20 Gy < 5%
Heart	Mean dose < 15 Gy
Liver	Mean dose < 25 Gy for right sided tumors; Mean dose < 1 Gy for left sided tumors
Ipsilateral kidney	Mean dose < 18 Gy
Contralateral kidney	Mean dose < 1 Gy
Esophagus	Mean dose < 34 Gy
Spinal cord	Max 45 Gy
Skin	Minimize hot spots and contour skin rind

### 3.4 Abdomen and pelvis tumor

Proton therapy for abdominal malignancies may lead to complications including fatigue, dermatitis, thrombocytopenia, gastrointestinal hemorrhage, diarrhea, duodenal perforation/ulceration, radiation‐induced liver injury (manifested as elevated transaminases, hyperbilirubinemia, or hepatic failure), biliary stricture, pleural effusion, rib fracture, radiation pneumonitis, ascending colon erosion, esophagitis, and colitis.

There remains a lack of established dose constraints for proton radiotherapy in hepatocellular carcinoma. At the PTCOG 2022 Conference Educational Session, referencing photon‐based QUANTEC guidelines, it was suggested that under standard fractionation: the Dmean to the liver (liver minus GTV) should be maintained below 28 Gy (RBE) for primary liver cancer and below 32 Gy (RBE) for liver metastases; for hypofractionated regimens, the hepatic Dmean should be limited to <13 Gy (RBE) (3 fractions) and <18 Gy (RBE) (6 fractions) in primary liver cancer, while thresholds of <15 Gy (RBE) (3 fractions) and <20 Gy (RBE) (6 fractions) are proposed for metastatic cases; notably, in Child‐Pugh B patients receiving primary liver cancer irradiation with single‐fraction doses of 4–6 Gy (RBE) across 3–5 fractions, a minimum spared normal liver volume of 700 cc should receive Dmean ≤15 Gy (RBE). Additional organ‐specific constraints are summarized in Table 8.^26^

**TABLE 8:** Dosimetric constraints for liver tumors stereotactic body proton therapy

OARs	Volume (cm^3^)	Volume maximum (GyE)	Point dose maximum (GyE)
Spinal cord	0.1	25	30
	<0.35	23	‐
	<1.2	14.5	‐
Stomach	0.1	27.5	32
	<10	18	‐
Duodenum	0.1	30	‐
	<5	18	‐
	<10	12.5	‐
Esophagus	<5	19.5	35
Small bowel	<5	19.5	35
Large bowel	<20	25	38
Lung total	1500	12.5	‐
	1000	13.5	‐
Kidney total	200	17.5	‐
Heart/pericardium	<15	32	38
Chest wall/rib	<30	30	43
Skin	<10	36.5	39.5

Proton therapy for pelvic malignancies may result in complications including hematologic toxicity secondary to bone marrow suppression, gastrointestinal toxicity, rectal bleeding, urinary irritative symptoms, sexual dysfunction, and radiation‐induced secondary malignancies. The monograph *Target Volume Delineation and Treatment Planning for Particle Therapy* has synthesized evidence from the RTOG 0529 trial^27^ to propose OARs dose constraints for anal canal proton radiotherapy, as outlined in Table 9. Furthermore, this comprehensive reference integrates findings from RTOG 0418^28^ and RTOG 1203^29^ trials to establish recommended dose limitations for gynecological malignancies, presented in Table 10.

**TABLE 9:** Recommended dose constraints to OAR when using proton beam therapy anal cancer

OARs	Recommended dose constraint
Small bowel	Max dose 54 Gy; 120 cc < 15 Gy
Bladder	50% < 35 Gy; 35% < 40 Gy; 5% < 50 Gy
Femoral heads	Max dose = 50 Gy; 50% < 30 Gy; 35% < 40 Gy; 5% < 44 Gy
Genitalia	50% < 20 Gy; 35% < 30 Gy; 5% < 40 Gy

**TABLE 10:** Recommended dose constraints to OAR when using Proton therapy for treatment of gynecologic malignancies

OARs	Dose constraints
Pelvic bone marrow	V10 GyE < 95%, V20 GyE < 76%
Large bowel	V40 GyE < 30% and V40 GyE < 300 cc
Small bowel	V40 GyE < 30% and V40 GyE < 300 cc
Kidney	V18 GyE < 66%
Bladder	V45 GyE < 35% or ALARA
Rectum	V40 GyE <60 or ALARA
Femoral heads	V30 GyE < 15%

### 3.5 Pediatric tumor

Pediatric cancer survivors achieving long‐term remission through standard therapies require heightened vigilance for late treatment‐related complications. Even low‐dose radiation exposure poses inherent risks to developing organs and tissues in children. Given the predominance of CNS malignancies in pediatric oncology coupled with proton therapy's dosimetric advantages in sparing organs at risk during craniofacial irradiation, the majority of clinical evidence derives from longitudinal follow‐up studies of proton‐treated CNS tumors.

Common toxicities following proton therapy for pediatric CNS tumors include headache, nausea/vomiting, cognitive impairment, cerebral necrosis, visual/auditory deficits, xerostomia, developmental delays, and secondary malignancies. In children/adolescents with CNS malignancies, treatment‐related complications may emerge incrementally over time, necessitating particular vigilance for late effects in survivors with favorable prognoses. Emerging evidence highlights the role of cerebellar and other brain substructures in neurocognitive function,^30^ suggesting that neurocognitive deficits may manifest as late complications in long‐term survivors of posterior fossa irradiation. Current consensus proposes brainstem dose constraints of D50 <52.4 Gy (RBE) and Dmax <60 Gy (RBE) for pediatric proton therapy.^31^ For posterior fossa tumors, particularly in surgically treated patients, adoption of more conservative dose limits is recommended.^32^ Institutional guidelines from Massachusetts General Hospital and MD Anderson Cancer Center delineate organ‐at‐risk constraints for posterior fossa proton therapy (Tables 11 and 12),^31^ though dose‐toxicity correlations for brainstem exposure remain unquantified.

**TABLE 11:** Dosimetric guidelines for OARs in pediatric proton treatment

OARs	DHV Point	Goal	Acceptable Range
Brainstem	Absolute dose at 0.1 cc; Absolute dose at 50% volume	<56.6 GyE; <52.4 GyE	56.6 GyE≤ D0.1cc < 58 GyE 56.6 GyE≤ D0.1cc < 58 GyE
Optic Chiasm / Optic Nerve	Absolute dose at 10% volume; Absolute dose at 0.1 cc	<55.4 GyE; <55 GyE	55.4 GyE≤ D10% < 56 GyE; 55 GyE≤ D0.1cc < 60 GyE
Cochlea	Mean absolute dose	<30 GyE	30 GyE≤ Dmean < 36 GyE
Retina	Absolute dose at 0.1 cc	<50 GyE	50 GyE≤ D0.1cc < 55 GyE
Lacrimal Gland	Mean absolute dose	<34 GyE	34 GyE≤ Dmean < 41 GyE
Spinal Cord	Absolute dose at 1 cc; Absolute volume at 50.4 Gy; Maximum absolute dose	<50.4 GyE; <5 cc; <54 GyE	50.4GyE ≤ D1cc < 52.2 GyE; ALARA; ALARA
Hippocampus Head	Mean absolute dose	<5 GyE	ALARA
Hippocampus Tail	Mean absolute dose	<20 GyE	ALARA
Hypothalamus	Mean absolute dose	<5 GyE	ALARA
Masticators	Relative volume at 40 Gy	<20%	ALARA
Mastoid	Mean absolute dose	<30 GyE	ALARA
Nasopharynx Posterior	Mean absolute dose	<30 GyE	ALARA
Brain	Relative volume at 115% dose	0%	ALARA
Brainstem Core	Absolute dose at 0.1 cc	<56.1 GyE	ALARA
Pituitary	Mean absolute dose	<30 GyE	ALARA
Scalp	Absolute volume at 30 Gy	<5 cc	ALARA
Temporal Lobe	Relative volume at 20 Gy	<10 %	ALARA
Circle of Willis	Estimated dose	<10 GyE	ALARA

**TABLE 12:** Normal tissue constraints applied at MD Anderson Cancer Center in clinical practice for posterior fossa targets

OARs	Regular constraints	Other constraints (Allowed constraints depending on tumor location and age)
Spinal Cord	Max dose≤51 GyE	V45GyE ≤ 1 cc V50.4GyE ≤ 1 cc Max dose ≤ 52 GyE
Brainstem	Max dose≤57 GyE V54GyE≤10 %	V60GyE ≤ 0.01 cc V55GyE ≤ 0.5 cc V30GyE ≤ 33%
Brain	V30GyE≤50%	‐
Chiasm	Max dose≤54 GyE	‐
Cochlea (Left/Right)	Max dose≤45 GyE Mean dose≤30 GyE	Mean dose≤38 GyE
Eye (Left/Right)	Max dose≤40 GyE Mean dose≤30 GyE	‐
Hippocampus (Left/Right)	D100% ≤ 9 GyE	‐
	Max dose≤ 16 GyE	‐
Lacrimal Gland	Max dose≤16 GyE Mean dose≤10 GyE	‐
Lens	Max dose≤ 5 GyE	ALARA
Mandible	Max dose≤70 GyE	‐
Optic Nerve	Max dose≤54 GyE	‐
Parotid Gland	Mean dose≤ 10 GyE	Mean dose≤ 15 GyE
Pituitary	Mean dose≤ 36 GyE	‐
Skin	Max dose≤66 GyE	‐
Temporal lobe	Max dose≤70GyE	ALARA
Thyroid	ALARA	‐

Pediatric head and neck cancer survivors treated with proton therapy require vigilant monitoring for long‐term complications including dental developmental abnormalities, craniofacial deformities, growth hormone deficiency due to pituitary dysfunction, hypothyroidism, visual/auditory impairments, and secondary malignancies.^33^ A Swedish national cohort study (2008‐2019) of 212 proton‐treated pediatric patients with CNS tumors, sarcomas, and germ cell tumors documented severe acute toxicities (Grade ≥3) in 25 patients affecting skin, mucosa, pharyngeal/esophageal regions, upper/lower gastrointestinal tracts, and ocular/auditory systems, while 15 patients developed significant late complications demonstrating temporal escalation in complication rates across multiple organ systems (skin/subcutaneous tissue, salivary glands, upper gastrointestinal tract, bone, joints, central/peripheral nervous systems, and sensory organs); notably, among 28 patients with 10‐year follow‐up, ≥21 exhibited at least one Grade 1–4 late complication and 14 experienced recurrent Grade 2–5 late toxicities.^34^

Current dose constraints for most OARs in pediatric proton radiotherapy remain undefined, particularly regarding dose‐related complications in thoracic, abdominal, and pelvic regions; while existing photon‐based radiotherapy experience^35^, ^36^ may serve as a provisional reference in the absence of proton‐specific clinical evidence, these constraints require adaptation to proton dosimetric characteristics with critical emphasis on maintaining dose‐volume parameters for key organs below established photon therapy thresholds. The limited availability of long‐term data in pediatric proton therapy research represents a significant ethical challenge. This issue arises from the relatively recent adoption of proton therapy for childhood cancers and the marked improvement in patient survival rates, which has resulted in insufficiently characterized data on late effects, such as secondary malignancies and developmental impacts on critical organs.

## 4 DISCUSSION

Unlike photons' exponential dose falloff, proton therapy's Bragg peak effect typically reduces distal OARs doses; however, clinical implementation must account for setup and range uncertainties to prevent Bragg peak misalignment near serial organs anterior or posterior to the target volume, necessitating stricter dose constraints and safety margins for organs distal to the tumor. Furthermore, despite the conventional application of a 1.1 RBE multiplier relative to photons, substantial uncertainties persist in proton RBE, which varies with linear energy transfer (LET), absorbed dose, tissue type, beam path characteristics, and physical parameters, potentially leading to divergent clinical outcomes compared to photon therapy. Particularly at the distal beam range, biological damage to OARs may exceed predictions based solely on physical dose metrics. Zhang et al.^37^ corroborated these findings through a retrospective analysis of 566 nasopharyngeal carcinoma patients receiving proton versus photon therapy, demonstrating higher temporal lobe injury incidence in the proton cohort (10% vs. 4%) with an estimated RBE of 1.18 for temporal lobe damage, underscoring the necessity for conservative dose constraint adjustments at distal beam ranges.

The variability in RBE also challenges proton dose constraints due to uncertainties in biological equivalence compared to photon models. While a fixed RBE of 1.1 is conventionally assumed, RBE is influenced by LET, tissue type, and clinical endpoints. For instance, high‐LET regions may elevate RBE beyond 1.1, increasing biological doses to serial organs like the temporal lobe, raising risks of late toxicities when photon‐derived constraints are applied. Additionally, inter‐patient variability complicates standardized limits, as RBE may amplify damage to developing organs. Current constraints often overlook these factors, risking under protection or overtreatment. Emerging strategies like LET‐based optimization aim to address this gap but require prospective validation. Integrating RBE variability into clinical guidelines is essential to optimize proton therapy's therapeutic ratio and safety.

Compared to photon beams, proton beams exhibit narrower lateral penumbras that enhance normal tissue sparing along the beam's lateral margins; however, their entrance channel demonstrates elevated dose deposition, potentially subjecting proximal normal tissues to increased radiation exposure risks, thus necessitating multi‐field optimization strategies to mitigate single‐beam toxicity in critical organs such as lungs, brain, and liver. While proton therapy offers dosimetric advantages, several unresolved debates require investigation. A prominent example is hippocampal sparing in adults with brain tumors, where theoretical benefits for neurocognitive preservation contrast with conflicting clinical evidence. Another critical debate centers on rib fracture risk in breast cancer patients: although proton therapy reduces heart and lung doses, its beam stopping patterns may increase anterior rib exposure. Additionally, proton dose distributions exhibit heightened sensitivity to respiratory motion compared to photon therapy, mandating rigorous motion management techniques including 4DCT simulation, respiratory gating, and robust adaptive planning to ensure dosimetric accuracy. Anatomical changes during treatment, such as tumor volume reduction or patient weight loss, may significantly alter proton beam range and dose distribution.^38^ For example, decreased tissue volume in shrinking tumors can lead to overshooting of the Bragg peak, potentially exceeding OAR tolerance doses. This phenomenon is particularly pronounced in thoracic and abdominal sites. Hence, repeat CT simulation and daily CBCT may reduce the anatomical uncertainty.

The clinical translation of proton therapy also faces multifaceted challenges, including substantial economic and technical barriers such as exorbitant infrastructure costs and operational complexities in beam delivery precision. Furthermore, limited robust clinical evidence from randomized controlled trials, particularly regarding survival benefits in common solid tumors. Evolving patient selection criteria, reflected in dynamic guideline revisions, further complicate clinical adoption due to unresolved controversies over anatomical versus biological stratification. These challenges collectively emphasize the need to balance proton therapy's theoretical physical advantages with pragmatic clinical and infrastructural realities.

While this analysis focuses on systematically characterizing the fundamental of proton dose constraints, the translation of these findings into standardized clinical protocols requires further validation through multi‐institutional trials and consensus‐building efforts. The established photon QUANTEC guidelines, derived from dose‐volume‐toxicity correlation models integrating cellular, animal, and clinical trial data, possess more robust evidence compared to current proton therapy standards. While emerging consensus and clinical studies have begun delineating relationships between OARs complications and proton dose constraints, these findings remain predominantly disease‐specific (e.g., lymphomas) and largely extrapolated from retrospective analyses or small‐scale cohorts, lacking validation through large‐scale multicenter prospective investigations. Owing to proton therapy's limited accessibility, high operational costs, and technical complexity, the accumulation of high‐quality clinical evidence progresses slowly, resulting in insufficiently validated dose‐volume‐toxicity correlations. Furthermore, substantial heterogeneity in equipment specifications, delivery techniques, and dosimetric protocols across proton centers complicates inter‐center comparisons and impedes reliable evidence generation. When proton‐specific toxicity data are unavailable, cautious adaptation of photon‐derived constraints with adjustments based on proton‐specific beam characteristics may serve as interim guidance. Future research necessitates multidisciplinary investigations combining radiobiological models (cellular/animal experiments), clinical validations, and mathematical simulations to establish proton‐optimized OARs constraints, while systematically accounting for clinical variables (genetic profiles, fractionation schemes, delivery technologies) through stratified analyses of complication types, temporal patterns, and severity gradients. Concurrently, international collaborative efforts via platforms like PTCOG, ASTRO, and ESTRO should prioritize standardized data collection and multi‐institutional trials to formulate consensus guidelines addressing proton therapy's unique physical and biological properties, particularly emphasizing beam path dependencies, standardized toxicity endpoints and variable RBE considerations at distal dose regions. We also advocated for an international registry to pool rare toxicity data across institutions.

## 5 CONCLUSION

This study identified critical distinctions between proton and photon therapy in OARs dose constraints: proton therapy necessitates comprehensive brainstem evaluation incorporating not only maximum point dose but also peripheral/central regional doses, with additional emphasis on volume‐dependent dosimetric parameters in pediatric cases. For non‐small cell lung cancer proton therapy, stringent constraints on lung V35‐V50 dose‐volume parameters are required, particularly recognizing V40≤23% as a significant predictor of radiation pneumonitis. These discrepancies stem from fundamental differences in the physical properties and biological effects between protons and photons.

Variations in recommended dose limits for the same organ across published studies. The variations in constraints primarily stem from heterogeneity in clinical contexts and methodological approaches across the literature. Firstly, differences in tumor types, disease stages, and patient age may lead to divergent tolerance thresholds for critical structures. Secondly, hypofractionated regimens versus conventional fractionation inherently alter biological dose effects, influencing dose constraints. The potential bias introduced by reliance on single‐institution studies and non‐peer‐reviewed conference proceedings should be acknowledged. However, in the absence of large‐scale clinical trial data, findings from single‐institution studies and non‐peer‐reviewed conference proceedings may serve as preliminary references rather than definitive conclusions.

## CONFLICTS OF INTEREST STATEMENT

The authors declare no conflicts of interest.

## Data Availability

The data that support the findings of this study are available on request from the corresponding author. The data are not publicly available due to privacy or ethical restrictions.
